# Heterostructured Electrocatalysts: from Fundamental Microkinetic Model to Electron Configuration and Interfacial Reactive Microenvironment

**DOI:** 10.1002/adma.202418146

**Published:** 2025-03-04

**Authors:** Yun Li, Md. Samim Hassan, Xin Zhao, Andrey L. Rogach

**Affiliations:** ^1^ Department of Materials Science and Engineering City University of Hong Kong 83 Tat Chee Avenue Kowloon Hong Kong SAR 999077 P.R. China; ^2^ IT4Innovations VSB – Technical University of Ostrava Ostrava‐Poruba 70800 Czech Republic

**Keywords:** adsorption energy theory, electronic state, heterostructured electrocatalyst, interfacial reactive microenvironment, microkinetic model

## Abstract

Electrocatalysts can efficiently convert earth‐abundant simple molecules into high‐value‐added products. In this context, heterostructures, which are largely determined by the interface, have emerged as a pivotal architecture for enhancing the activity of electrocatalysts. In this review, the atomistic understanding of heterostructured electrocatalysts is considered, focusing on the reaction kinetic rate and electron configuration, gained from both empirical studies and theoretical models. We start from the fundamentals of the microkinetic model, adsorption energy theory, and electric double layer model. The importance of heterostructures to accelerate electrochemical processes via modulating electron configuration and interfacial reactive microenvironment is highlighted, by considering rectification, space charge region, built‐in electric field, synergistic interactions, lattice strain, and geometric effect. We conclude this review by summarizing the challenges and perspectives in the field of heterostructured electrocatalysts, such as the determination of transition state energy, their dynamic evolution, refinement of the theoretical approaches, and the use of machine learning.

## Graphical abstract

Heterostructures have emerged as advanced electrocatalysts to convert earth‐abundant simple molecules into high‐value‐added products. On the basis of atomistic understanding to accelerate the electrochemical processes, the architecture of heterostructured electrocatalysts is comprehensively discussed from the point of view of modulating electronic configuration and interface reactive microenvironment. The influence of rectification, space charge region, built‐in electric field, synergistic interactions, lattice strain, and geometric effect is considered.

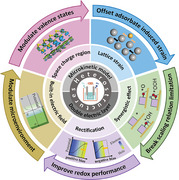

## 1 Introduction

Over the last decade, a slight increase of 0.25 °C in global temperature has resulted in the rise of extreme weather events, causing significant adverse societal costs amounting to ≈143 billion USD per year.^[^ ^1^ ^]^ This increase is largely attributed to anthropogenic greenhouse gas emissions, which is pushing society to ensure a rapid transition from reliance on fossil fuels to renewable electricity. Electrochemical processes, which can transform common molecules (e.g., CO_2_, H_2_O, N_2_) into high‐value products (e.g., hydrocarbons, hydrogen, ammonia), offer a feasible way to reduce greenhouse gas emissions. Electrocatalysts play a key role in these energy conversion processes, including carbon dioxide reduction (CO_2_RR), hydrogen evolution/oxidation reaction (HER/HOR), oxygen evolution/reduction reaction (OER/ORR), and nitrogen reduction reaction (NRR), as they improve the rate, efficiency, and selectivity of the chemical transformation involved.^[^ ^2^ ^]^

Chemical reactions typically include multiple steps with continuous breaking and reconstruction of chemical bonds, which are related to the coupling of atomic orbitals and the rearrangement of electrons. The intrinsic activity of catalysts, which is quantified as the reduction of the activation energy barrier, is strongly influenced by interactions of their valence electrons with participating substances.^[^ ^3^ ^]^ In the electrochemical process, there is a continuous electron drift within the catalyst, as well as a mass transport at the interface of the electrode and electrolyte. Hence, electrocatalysts need to have high conductivity and there should be fast transport of both the reactants and products. Heterostructures, which we define here as materials consisting of at least two either physically (through the Van der Walls’ forces) or chemically bound components, not only provide individual functions of their single constituting phases, but also exhibit tunable electronic states at the interface. As an example, Pd‐PdO nanodomains on the Ru metallene oxides,^[^ ^4^ ^]^ γ‐FeOOH‐NiOOH,^[^ ^5^ ^]^ and Ru‐CrO_x_ cluster‐cluster junctions,^[^ ^6^ ^]^ showed great potential in electrocatalysis, stimulating favorable electron redistribution and thus facilitating electrochemical reduction and oxidation reactions.^[^ ^7^ ^]^ Heterostructure engineering can significantly improve the conductivity of electrocatalysts, under which the drop in Fermi levels pushes electrons across the interface and strengthens the electron drift.^[^ ^8^ ^]^ Simultaneously, the surface polarization caused by the accumulated charge carriers can also accelerate the transport of reactants and products via favorable electrostatic interactions.^[^ ^9^ ^]^ Compatibility of different components at the interface ensures the ability to break the scaling relation limitation of adsorption energies for intermediates by eventually changing the reaction mechanism, thus lowering the overpotential for electrochemical processes.

It is hard to suggest a standard manual for engineering electrocatalysts with optimized electronic states. However, combining experiments and theoretical modeling offers the potential to gain an atomistic understanding of the electrocatalytic processes, which is helpful for designing active catalytic sites. Starting from the late 19th century, it was experimentally found that the reaction rate is proportional to the reaction constant (*k*) and the power function of reactants’ concentration. The Arrhenius equation ($\frac{d\;lnk}{d\;T}=\frac{E_{a}}{RT^{2}}$) indicates that the reaction constant is determined by activation energy (*E_a_*), which is closely associated with the exchange current density in the electrochemical process. The Sabatier theory pointed out that a modest binding of intermediates on the catalyst surface, neither too strong nor too weak, facilitates their adsorption and desorption. For example, a “volcano” plot drawn by Sergio^[^ ^10^ ^]^ correlates the cathode exchange current density of hydrogen evolution for water electrolysis with the chemisorption energy of hydrogen. The rise of density function theory (DFT) bridged electronic states of electrocatalysts and intermediates with their binding strength, corresponding to the total energy change before and after the hybridization of atomic orbitals and the reconfiguration of valence electrons. In this sense, the term “*electronic state*” can be used as a good descriptor for the intrinsic activity of catalysts.

Still, the process of screening optimized electrocatalysts remains a challenge, as it requires atomic‐scale structural models able to encompass multiple length and time scales and modification of local reactive microenvironment.^[^ ^11^ ^]^ In this review, we will first discuss the state‐of‐the‐art of fundamental microkinetic model, adsorption energy theory, and electric double layer model. On that basis, we will discuss the relation between the reaction rate, binding strength of intermediates, valence states of active sites, and the interfacial reactive microenvironment between electrode and electrolyte. We will emphasize the potential of heterostructured electrocatalysts and consider the most important characteristics of the commonly used heterostructures, including rectification, space charge region, built‐in electric field, synergistic effect, as well as lattice strain, which allow us to modulate the electron configuration of active sites and the microenvironment where the reaction occurs. Besides the electronic characteristics, the well‐designed geometry of heterostructured electrocatalysts could also enhance the electrochemical performance through accelerating reaction kinetic. We conclude this review by summarizing the challenges and perspectives in the field of heterostructured electrocatalysts, such as the determination of transition state energy, their dynamic evolution, refinement of the theoretical approaches, and the use of machine learning.

## 2 Fundamental Theories of Electrocatalysis

Consideration of the structure‐function relation is an important aspect of the development of optimized electrocatalysts. The Sabatier empirical principle, stating that the proper adsorption of intermediates, neither too strong nor too weak, offers the highest activity to the catalyst, remains an important concept in determining ideal electrocatalysts. Based on the Brønsted‐Evans‐Polanyi (BEP) relationship and the scaling relation of intermediates for binding strength in one reaction, the microkinetic model developed by Nørskov and co‐workers^[^ ^12^ ^]^ offered a quantitative consideration for the relation between the kinetic rate and the adsorption energy of a specific intermediate. Importantly, the development of the adsorption energy theory revealed the relation between the valence state of catalysts and the adsorbing strength, thus guiding the screening of the “perfect” catalysts.

### 2.1 Microkinetic Model

Following Sabatier's empirical principle, catalysts with a moderate adsorption energy of intermediates in the multiple‐step catalytic reaction would experience high performance.^[^ ^13^ ^]^ Once the binding strength is too strong, it becomes hard for the intermediates to desorb, leading to the poisoning of the catalyst surface; on the other hand, the binding that is too weak makes it difficult for intermediates to adsorb at the surface. While intuitively clear, Sabatier's principle was lacking a theoretical kinetic model able to quantify and construct the connection of the binding energy and reaction rate – the so‐called “volcano” map. That's why, the “trial‐and‐error” approach has been commonly used to determine a proper catalyst for quite a long time. The emergence of the microkinetic model and the recent widespread use of DFT based approaches have enabled more efficient, computer‐based catalyst design.^[^ ^14^ ^]^ The microkinetic model, which connects the atomic scale phenomena to macroscopic observables, has been developed based on several important contributions, namely the power law rate expression, the transition state theory, and the discovery of the BEP relationship.

The kinetic rate can be determined as a first approximation of a power law rate expression for the experimental data, as it is associated with the reactant concentration and the rate constant.^[^ ^15^ ^]^ The collision theory states that the rate constant for the reaction among reactants is associated with the *activation energy*.^[^ ^16^ ^]^ The transition state theory further postulates the existence of the so‐called *transition state*, which clarifies the definition of activation energy and allows for the incorporation of the molecular structure into the rate constant estimation.^[^ ^17^ ^]^ For elementary electrochemical reactions with one electron transfer,

(1) ∗A+B±e→∗C+D

the electrochemical kinetic rate of the surface reaction per unit surface area, *r*, is:

(2) $$r=k\theta_{A}\;a_{B}=\frac{k_{B}T}{h}\;\exp\left(-\frac{\Delta G}{RT}\right)\theta_{A}a_{B}$$

(3) $$\Delta G=\Delta G^{0}\pm \beta \eta F\;$$

where *θ_A_* represents the fractional coverages of species *A* adsorbed at the surface; *a_B_* is the activity of species *B* near the electrode surface; $\Delta G^{0}$ is the standard Gibbs free energy difference for the formation of the transition state; *β* is the symmetry factor reflecting the degree of change in free energy derived by electrode potential; and *η* is the overpotential applied on the electrode. Hence, the key point to designing highly efficient electrocatalysts is to modulate the adsorption energy of the intermediate.

Most of the electrochemical reactions involve multiple steps, which determine a large diversity of intermediates.^[^ ^18^ ^]^ Thus, it is hard to speculate about the structure of the transition state and its minimum potential energy as the product of multiple possibilities of reaction paths and types of transition states. Moreover, due to the extremely short lifetime (picoseconds), it is still challenging to detect and analyze spectroscopic data for transition states. To date, resonances spanning the transition state range have only been distinguished for simple atom‐diatom reactions, such as *F + HI* = *HF + I* and *F + H_2_ = HF + H*, which was done through anion photoelectron spectroscopy.^[^ ^19^ ^]^ No such data has been obtained for polyatomic reactions, leaving their impact on the dynamics of these systems unexplored.^[^ ^20^ ^]^ This bottleneck severely limits the detection of transition states and the determination of the related activation energies.

The BEP relationship states that the activation energy (*E_a_*) scales with the reaction energy (*E_r_*), through the equation:

(4) $$E_{a}=\alpha E_{r}+\beta$$

To determine the reaction energy, a large statistics of the ground state energies for different types of adsorbates on a large amount of catalytic surfaces is required.^[^ ^21^ ^]^ This necessitates the development of theoretical methods to calculate such large quantities in an effective and accurate manner so as to treat the complex systems of interest and solve the multiple‐electron Schrödinger equations for heterogeneous catalysis. Benchmarking theoretical calculations against experimental measurements is necessary, and indeed the developed computational methods can provide adsorption energies within an acceptable error. The advent of the generalized gradient approximation methods, such as Lee‐Yang‐Parr (LYP) functional and Perdew–Burke–Ernzerhof (PBE) functional, delivered ground state energies for intermediates with an acceptable error of 0.1–0.2 eV through DFT calculations.^[^ ^22^ ^]^ Similar to the BEP relationship, a correlation between adsorption energies of the transition state and intermediates in an elementary reaction across a variety of catalytic surfaces also exists.^[^ ^23^ ^]^ For instance, the energy of the transition state for the N_2_ molecule is related to the dissociative chemisorption energy of atomic nitrogen. On the basis of this scaling relation, Nørskov developed the microkinetic model, which associates the reaction rate with the binding energies.^[^ ^24^ ^]^ For an elementary reaction *i* with *j* species, the rate constant *k* for electrocatalysis can be expressed as:

(5) $$k=k_{i}^{\prime }\;\exp\left(-\frac{\alpha \left(\sum_{j}v_{j,i}BE_{j}\right)\pm \beta \eta F}{RT}\right)$$

where $k_{i}^{\prime }$ is a rate constant that remains irrespective of the change of the binding energies for the intermediates, α is the slope of the BEP Equation (4), *v*_*j*,*i*_ is the stoichiometric coefficient, and *BE_j_* is the binding energy of intermediate *j*.

The microkinetic model, combined with thermodynamic binding energies calculated by DFT, can determine the active and non‐active phases of electrocatalysts under an applied bias by comparing them with experimental data. Pourbaix diagram for the Co‐H_2_O system illustrates the phase evolution of *β*‐Co(OH)_2_ in relation to the increasing oxidation potential, which orderly transforms to *β*‐CoOOH and CoO_2_. On the basis of steady state microkinetic model, William and coworkers^[^ ^25^ ^]^ separately calculated the OER polarization current curves of $\left[11\bar{2}0\right]$ plane in *β*‐Co(OH)_2_, *β*‐CoOOH and CoO_2_, considering the variation of original and derived phases under increasing potential. They found that the simulated current density of *β*‐Co(OH)_2_ decreased rapidly at high overpotential, which was not observed experimentally. In contrast, the simulated data for *β*‐CoOOH and CoO_2_ aligned closely with experimental findings, indicating that *β*‐Co(OH)_2_ is inactive, while *β*‐CoOOH and CoO_2_ are potentially active phases for the OER. Besides, recent advancements in artificial intelligence and machine learning made the microkinetic model more efficient in identifying mechanisms for multiple electron transfer reactions.^[^ ^26^ ^]^ Cheng and coworkers^[^ ^27^ ^]^ developed a microkinetic‐guided machine learning method to identify the lowest‐energy pathway of CH_4_ synthesis by the hydrogenation of CO_2_ and CO. Stochastic surface walking‐based reaction sampling method was employed to simultaneously explore possible elementary reactions on Cu [111], Cu [211], 0.11 ML Zn‐Cu [211], and 0.22 ML Zn‐Cu [211] starting from CO_2_ + H_2_, HCOOH + H_2_, HCHO + H_2_, HCOO* + CO_2_ + H_2_, HCOO* + HCHO + H_2_, and HCOO* + HCOOH + H_2_. These reactions were determined based on the lowest free energy barrier of their respective transition states. By integrating each derived elementary step from these branches into a comprehensive reaction network, the lowest‐energy pathway was identified following the formate mechanism: CO_2_ → HCOO* → HCOOH* → H_2_COOH* → HCHO* → CH_3_O* → CH_3_OH* → CH_3_OH.

### 2.2 Adsorption Energy Theory

The adsorption energy of the key intermediate, related to the rate constant of the reaction, can be regarded as a descriptor for evaluating the activity of the catalyst. Nørskov^[^ ^28^ ^]^ found out the scaling relations of adsorption energies among AH_x_ (A = C, N, O, S) adsorbates regardless of the catalysts, meaning that the adsorption energies of AH_x_ species can be calculated through the scaling equation with those known for A species. Similar scaling relations were found for other monodentate adsorbates with comparable molecular structures and electronic configurations, particularly for intermediates belonging to one reaction, such as *CO, *COH, and *COOH within electrocatalytic CO_2_ reduction.^[^ ^29^ ^]^ Consequently, the golden rule for designing an optimal catalyst is to obtain appropriate binding energies for the key species participating in a rate‐determining step.

**Figure 1:**
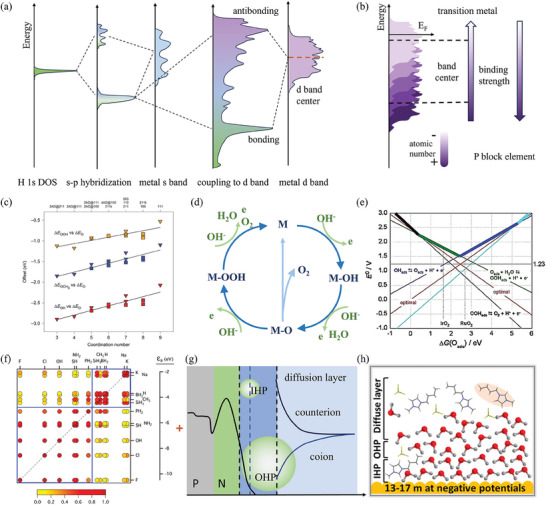
a) Schematic illustration of the change in the local electronic structure at a hydrogen atom upon its adsorption on transition metal surfaces. b) Upshift of the *d* band or *p* band center of atoms with decreasing atomic number in the same period, and the relative binding strength. c) Coordination number – energy relations for atomic oxygen and oxygenates; Reproduced with permission.^[^ ^33^ ^]^ Copyright 2015, Springer Nature. d) Schematic illustration of the OER mechanism in alkaline solution; e) Equilibrium potentials of the elementary reactions versus free binding energy of oxygen on metal oxides Δ*G*(O_ads_); Reproduced with permission.^[^ ^34^ ^]^ Copyright 2011, Elsevier. f) Linear correlations between the adsorption energies of various adsorbates, arranged by their HOMO energies; Reproduced with permission.^[^ ^35^ ^]^ Copyright 2014, American Chemical Society. g) Illustration of the double electric layer under positive bias. h) Schematic diagram of the water network in a non‐aqueous [BMIM][BF_4_] ionic liquid. Reproduced with permission.^[^ ^36^ ^]^ Copyright 2023, American Chemical Society.

For transition metals, the *d* band theory points out that the *d* band states, including the filling state, band width, and the *d* band center, affect the adsorption energy through the hybridization with molecular orbitals of intermediates. As seen in **Figure** **1**a, the sharp atomic states of the adsorbing molecule become broaden into resonance and shift downward after hybridizing with the metal *sp* band. Subsequently, these renormalized states couple with the metal *d* band and split into covalent bonding and antibonding states below and above the initial states of the adsorbing molecule and metal respectively. While moving further to the left part of the periodic table for the 3*d*, 4*d*, or 5*d* metals, their *d* band center shifts toward the Fermi level, and the increased mixing adsorbate‐*d* antibonding states become more depopulated, which results in a stronger binding of the adsorbates (Figure 1b).^[^ ^30^ ^]^ For the *p*‐block element active sites, such as C, N, etc., a similar *p* band model was established, where the hybrid system with a higher *p* band center yields weaker binding, contrary to the trend of the *d* band.^[^ ^31^ ^]^ Such discrepancy can be ascribed to the fact that the antibonding states for the adsorbate and *p*‐block element are almost fully occupied, as only a few electron states are available in their conduction bands.^[^ ^32^ ^]^

However, these band theories largely overlooked the geometry of the catalyst and the relative preference of intermediates, which serves as a more direct descriptor for the design of the catalyst. Different adsorbing sites, such as atop, bridge, and hollow sites (face center cubic and hexagonal closed‐packed), exhibit different binding sensitivity. For example, Papoain et al.^[^ ^37^ ^]^ showed that methyl and ethyl groups bind more strongly on the top site of the Pt (111) facet, as compared with the bridge site and face center cubic site. The coordination number (CN) and the relative “coordination‐activity” plot were further used by Bandarenka and co‐authors to predict the geometric structure for the optimal active site.^[^ ^38^ ^]^ As a result of the finite‐size effect, the CN of a nanoparticle differs from the extended surface with the same facet, and thus they applied a generalized coordination number ($\bar{\mathrm{CN}}$) by weighing each first‐nearest neighbor atom to replace the conventional CN. As seen in Figure 1c, the existence of scaling relation between $\bar{\mathrm{CN}}s$ of Pt atoms at the surface and adsorption energies for *OH and *OOH species have been demonstrated, and a volcano plot linking $\bar{\mathrm{CN}}s$ and the potentials of the two rate‐determination steps were established, showing that Pt nanoparticles with $\bar{\mathrm{CN}}=8.3$ offers the most optimal ORR activity. Additionally, the lattice parameter, reflecting the degree of overlapping of atomic orbitals and the repulsion of the electron cloud, also determines the catalyst's ability to bind with reactive species. For example, platinum‐lanthanide electrodes (Pt_5_M, where M is La, Ce, Sm, Gd, Tb, Dy or Tm), whose covalent radius enlarges with increasing atomic number, demonstrated a volcano plot for the kinetic current density as a function of the lattice parameter for the ORR process.^[^ ^39^ ^]^

### 2.3 Limitation of Linear Scaling Relation

Regardless of the binding strength of the adsorbates, the kinetics of catalytic processes are usually restricted by the *linear scaling relation* (LSR): when a catalyst binds one particular adsorbate more strongly, it tends to bind similar adsorbates either stronger or weaker. This scaling relation reduces the degree of freedom for calculation of multiple proton‐electron transfer reactions; however, it also causes the existence of hard‐to‐reach optimal adsorption with neutral chemisorption energy, for the cases of oxygen evolution/reduction reactions, electrocatalytic CO_2_ reduction, nitrogen reduction, Buchwald‐Hartwig amination reaction, etc.^[^ ^40^ ^]^

The LSR describing the relation among the binding energies of different intermediates at the same transition metal surface can be formulated as:

(6) $$\Delta \;E_{2}^{\left(i\right)}=A_{1,2}^{\left(i\right)}\;\cdot \Delta E_{1}^{\left(i\right)}+B_{1,2}^{\left(i\right)}$$

where $\Delta E_{1}^{\left(i\right)}$ and $\Delta E_{2}^{\left(i\right)}$ are binding energies for species 1 and 2 adsorbing onto the lattice facet (i). For monodentate adsorbates on close‐packed or low‐index metal surfaces, the slope ($A_{1,2}^{\left(i\right)}$) strongly relies on the valence state of the adsorbed atom.^[^ ^28^, ^41^ ^]^ The offset ($B_{1,2}^{\left(i\right)}$) has been proved to explicitly depend on the structure of the active site: when the surface CN for certain ranges (typically CN ≥ 3) decreases, the corresponding adsorption energy was found to become more negative.^[^ ^33^ ^]^ When the structure‐dependent offset $B_{1,2}^{\left(i\right)}$ remains constant, it is not possible to achieve a moderate energy profile for two or more intermediates because of the trade‐off LSR relation defined by the similar valence states.

The OER undergoing an adsorption energy mechanism is illustrated in Figure 1d, where the LSR exists among involved oxygenated intermediates. The average LSR slope of all facets of transition metals is 0.51 for *OH versus *O and 0.54 for *OOH versus *O since O lacks two electrons while O in the form of OH and OOH needs one electron to keep its stable 8‐electron structure (Figure 1e).^[^ ^33^ ^]^ The ideal energy difference between *OH and *OOH should be 2.46 eV, however, the existence of LSR causes a larger difference, namely 3.2 ± 0.2 eV, resulting in a fundamental additional overpotential of (3.2 ± 0.2−2.46) (eV)/2 *e* ≈ 0.27–0.47 V.^[^ ^34^, ^42^ ^]^

Scaling laws for the hydrogen‐contained molecule (AH_x_, A = C, N, O, S) were explained using the *d* band model and the effective medium theory.^[^ ^28^ ^]^ The *d* band model ascribes the adsorption energy to hybridization ($\Delta E_{d}^{hyb}$) and orthogonalization ($\Delta E_{d}^{orth}$, Pauli repulsion), which scales with the square of the Hamiltonian matrix element between the adsorbate and the metal *d* states ($V_{ad}^{2}$).^[^ ^30^ ^]^ According to the effective medium theory, each surface atom contributes roughly equal electron density to the adsorbing A species, which is in line with the value of (*x_max_* − *x*)/*x_max_*, where *x_max_* is the maximum number of hydrogen atoms bound with atom A. Because the Hamiltonian matrix $V_{ad}^{2}$ has a positive correlation with the *d* band electron density for transition metal according to the *d* band model, the adsorption energies for AH_x_ species are in line with each other. However, this model can't explain why LSR relation exists among adsorbed atoms inside and outside the same group of the periodic table of elements. Medlin and coauthors^[^ ^35^ ^]^ rationalized the grouping of the adsorbates based on electronic structure:

(7) $$E_{ads}=E_{elec}+a_{1}V_{ad}^{2}f+a_{2}V_{ad}^{2}$$

where *E_elec_* reflects the changes in the metal bands and the absorbate's highest occupied molecular orbital (HOMO); *a*_1_ and *a*_2_ are constants, associated with the type of pined atom of the adsorbate; and *f* is related to the *d* band filling state. The authors found out that monovalent adsorbates with a similar high or low HOMO energy experienced scaling correlations of adsorption energies, which led to the classification of those adsorbates into two groups as illustrated in Figure 1f. And the *d* band filling state determines the strength of the binding energy between the adsorbate and metal surface.

To address the limitation of scaling relation in multiple electron transfer reactions, taking the OER process as an example, one approach is to consider the mechanism that allows for the coupling of adjacent *O and averting the proton‐electron transfer process for *OOH.^[^ ^43^ ^]^ A recently proposed mechanism that involves the lattice oxygen participating in the OER process considers a direct *O coupling to avoid producing any *OOH intermediate species.^[^ ^44^ ^]^ The similarity of HOMO states driven scaling relation between *OH and *OOH prevents the isolated active site from being an ideal catalyst, combining energetic features of each component toward one of the critical oxygen‐containing intermediates, the well‐designed architecture of heterostructures has the capability to break LSR.^[^ ^45^ ^]^

### 2.4 Electric Double Layer

Chemical reactions are accompanied by the gain and loss of electrons, which occurs at the surface of a catalyst. The catalyst's performance is thus directly influenced by its surface state. In an electrochemical reaction, the external voltage first charges the electrode, which is thereafter screened by the Helmholtz layer and diffusion layer formed by the transfer and adsorption of ions with opposite charges in the electrolyte (Figure 1g). When the applied voltage stimulates the electron transfer across the electrode, the potential equilibrium at the interface of the electrode and the electric double layer becomes broken. The difference in mobility between electrons and ions at the interface results in the inadequate screening of the surface electric field. This leads to changes in both structure and potential within the electric double layer, ultimately affecting the physicochemical properties of the space charge region.^[^ ^46^ ^]^

Taking hydrogen evolution reaction in the alkaline medium as an example, when the electrode is negatively charged, sodium/potassium ions from the base are primarily accumulated at the outer Helmholtz layer, keeping the first hydration shell intact because of the weak dispersive attraction toward the electrode. The Helmholtz potential drives the so‐called H‐down orientation of water within the inner Helmholtz layer, and simultaneously the hydrogen bond network connectivity keeps water molecules in the same H‐down orientation in the outer Helmholtz layer. As a result, a surface‐normal macroscopic dipole of water layers is generated, which influences the energy of the transition state.^[^ ^47^ ^]^ Experimental evidence showed that the intensity of the O–H vibration peak centered ≈3000 cm^−1^ in the water layer decreased as the potential drops from 0.5 to 0 V. This was accompanied by a slight growth in the intensity and a blue‐shift of the ν(O–H) band between 3300 and 3500 cm^−1^. These spectral changes were attributed to the potential‐dependent re‐orientation of the H‐up water toward H‐down water.^[^ ^48^ ^]^

However, since in the case of HER water molecules act both as reactant and solvent in an aqueous system, it is hard to distinguish the state of medium and interfacial water and their influence on HER performance. Jiang and coworkers^[^ ^36^ ^]^ replaced water with 1‐butyl‐3‐methylimidazolium tetrafluoroborate ionic liquid ([BMIM][BF_4_]) as a solvent. As shown schematically in Figure 1h, the increasing molarity of water molecules in the [BMIM][BF_4_] solvent resulted in a gradually appearing hydrogen bond net, as evidenced by the blue shift and broadening of the O–H vibration peak detected by the surface‐enhanced infrared absorption spectroscopy. It was also found that the HER activity depended on the form of an asymmetric four‐coordinated water network, which was confirmed by the introduction of hydrophilic Li^+^ and hydrophobic tetrabutylammonium cations. The former cations disrupted the water network in the electric double layer via strong ion‐dipole interactions, leading to restricted HER activity; while the later enhanced connectivity of the hydrogen bond network, thus enhancing HER activity. Similarly, it was reported that the water network in the basic medium became disturbed electrostatically due to the presence of OH^−^ anions, which were attracted by accumulated cations (Na^+^ or K^+^) in the outer Helmholtz layer.^[^ ^49^ ^]^ Jia and colleagues^[^ ^50^ ^]^ proposed that the hydrogen bond network was involved in the HER process by facilitating the transfer of the adsorbed hydroxyl (*OH) on active sites, which was produced through the dissociation of water molecules in a basic medium. Hydroxyls received protons from the hydrogen bonded water molecules in the second water layer and finally diffused into the bulk electrolyte. Additives like caffeine and N‐methylimidazole with negatively charged pyridinic nitrogen were found to promote the HER rate by forming stronger hydrogen bonds and enhanced the diffusion of hydroxyl without the need for any additional steps for adsorption and desorption at the catalyst surface.^[^ ^51^ ^]^ However, if the hydrogen bonds are too strong, they may increase the energy cost to adapt their configuration to stabilize the activated complex participating in HER.^[^ ^52^ ^]^

## 3 Rectification of Heterostructured Electrocatalysts

Heterostructures possess an interface between two different components/phases, whose existence provides specific properties to electrocatalysts, different from those of single components. Once the two components come into contact, charge redistribution near the interface will start spontaneously until the Fermi levels reach equilibrium. As a result, electrons and holes would gather separately around the two sides of the interface, forming the *built‐in electric field* (BIEF). After applying positive or negative bias, BIEF undergoes specific dynamic evolution for the cases of Schottky junctions, *p‐n* junctions, *p‐p* junctions, *n‐n* junctions, and metal–metal junctions, which is known as the *rectification effect*. This effect plays an important role in accelerating the electrochemical process. **Table** **1** shows some typical examples of heterostructured electrocatalysts used for water electrolysis. In the following discussions, we will consider these examples one‐by‐one.

**Table 1:** Examples, work functions, and water electrolysis performance for heterostructured electrocatalysts based on Schottky junctions, *p‐n* junctions, and *p‐p* junctions.

Junction	Heterostructure's components	Work Function [eV]		Overpotential [mV]		Refs.
		Metal	*n*‐type	HER	OER	
Schottky	MoB/g‐C_3_N_4_	MoB	g‐C_3_N_4_	133	–	[53]
			−7.3			
	Co/CoSe	Co	CoSe (*p*‐type)	–	335	[54]
		−5	−5.66			
	Ni‐W_5_N_4_|NiFeOOH	Ni‐W_5_N_4_	NiFeOOH		216	[55]
		−5.41	−3.73			
	P‐CoFe‐LDH@Mxene	Mxene	P‐CoFe‐LDH	85	252@100 mA cm^−2^	[56]
		−	−			
	Co/MoC	Co	MoC	92	279	[57]
		−	−			
	Ni/NiFe‐LDH	Ni	NiFe LDH	56	160	[58]
		−6.26	−4.74			
	NiFe(OH)_x_/Ni_3_S_2_	Ni_3_S_2_	NiFe(OH)_x_		209@100 mA cm^−2^	[59]
		−5.91	−4.97			
	NiSe_2_/MoSe_2_	NiSe_2_	MoSe_2_	79		[60]
		−4.93	−5.07			

Junction	Heterostructure's components	Work Function (eV)		Overpotential (mV)		Refs.
		*p*‐type	*n*‐type	HER	OER	
*p‐n*	CuS@MoSe_2_	CuS	MoSe_2_	72	219	[61]
		−6.26	−4.05			
	Co_2_N/CoP	Co_2_N	CoP	44	227	[62]
		−5.52	−3.92			
	CLDH/CP	Cu_3_P	Co LDHs	111	220	[63]
		−6.03	−4.25			
	Ni_2_P/CuCo_2_S_4_	CuCo_2_S_4_	Ni_2_P	183	360@40 mA cm^−2^	[64]
		−6.06	−4.6			
	CoP/CeO_x_	CoP	CeO_x_	118	−	[65]
		−3.93	−5.56			
	MnCo‐CH@NiFe‐OH	MnCo‐CH	NiFe‐OH	177	186	[66]
		−4.75	−4.01			

Junction	Heterostructure's components	Work Function (eV)		Overpotential (mV)		Refs.
		*p*‐type	*p*‐type	HER	OER	
*p‐p*	NiSe_2_/FeSe_2_	NiSe_2_	FeSe_2_	−	256	[67]
		−6.88	−6.36			
	CoP‐CoO	CoP	CoO	104	210	[68]
		−5.46	−5.35			

Note: except as otherwise specified, all overpotentials are at 10 mA cm^−2^

### 3.1 Schottky Junctions

**Figure 2:**
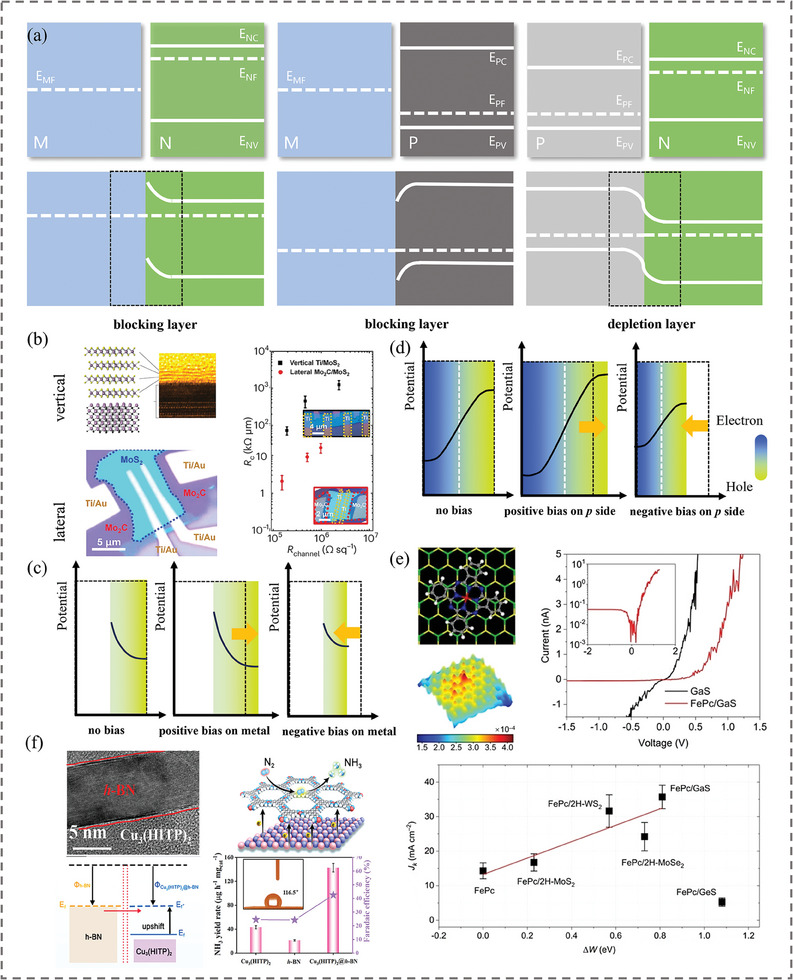
a) Energy diagrams of heterostructures formed by metal and *n*‐type semiconductor, metal and *p*‐type semiconductor, and *p*‐type semiconductor and *n*‐type semiconductor. b) Contact and sheet resistance for vertical Ti/MoS_2_ and lateral Mo_2_C/MoS_2_ heterostructures. Reproduced with permission.^[^ ^75^ ^]^ Copyright 2018, American Chemical Society. Potential profiles at the interface of c) metal and *n*‐type semiconductor, d) *p*‐type semiconductor and *n*‐type semiconductor after applying bias. e) Rectification effects of *p*‐type FePc on GaS, H‐MoS_2_, 2H‐MoSe_2_, 2H‐WS_2,_ and *p*‐type GaS. Reproduced with permission.^[^ ^76^ ^]^ Copyright 2022, Wiley‐VCH. f) *n‐n* type heterojunction of Cu_3_(HITP)_2_@h‐BN and its performance in the NH_3_ reduction compared with bare Cu_3_(HITP)_2_ and h‐BN. Reproduced with permission.^[^ ^77^ ^]^ Copyright 2023, Wiley‐VCH.

When the metal is in tight contact with a semiconductor component (which can be *n*‐type or *p*‐type), Schottky or ohmic contact is formed. The work function of the metals varies from the lowest value of 1.93 eV for Cs to the highest value of 5.36 eV for Pt, changing systematically upon an increase in the atomic number. In a heterostructure formed by metal and *n*‐type semiconductor (**Figure** **2**a), when the work function of the metal is larger than that of the semiconductor, electrons would flow from the semiconductor to the metal until the arising potential drop is enough to compensate for the difference of Fermi energy levels. This results in an upward bending of the conduction band (CB) and valence band (VB) in the *n*‐type semiconductor, which creates a blocking layer with high resistance at the interface, where the density of charge carriers is higher than in the bulk (we use the word “bulk” here to distinguish this extended region of the (semiconductor) component from the interface).^[^ ^69^ ^]^ Semimetals, such as transition metal dichalcogenides like 1T’‐WS_2_, 1T’‐MoS_2_, 2H‐NbS_2_, and tr‐BeN_4_,^[^ ^70^ ^]^ as well as some members of the carbon family, like graphene,^[^ ^71^ ^]^ carbon nitride,^[^ ^72^ ^]^ and carbon nanotubes,^[^ ^73^ ^]^ can also form Schottky junctions with semiconductors. In such 2D heterostructures, quantum confinement of electrons may lead to edge and surface thermal emissions, which reflects their two kinds – lateral and vertical Schottky junctions.^[^ ^74^ ^]^ Ang et al.^[^ ^75^ ^]^ proposed a universal scaling law in the form of the Arrhenius equation (ln(*J_TE_*/$T_{e}^{\beta }$) vs $T_{e}^{-1}$) to consider the electron thermal emission of 2D Schottky junctions. According to this study, the emission and the contact dynamics can be accurately determined by replacing *β* = 2 for metal with 3/2 for lateral and with 1 for vertical semimetal‐based Schottky junctions. The lateral contact structure of the Mo_2_C/MoS_2_ junction exhibited a lower Schottky barrier height than the vertical Ti/MoS_2_ one, as illustrated in Figure 2b.

The width of the space charge region is associated with the applied voltage; when the external electric field is aligned with the BIEF direction, it pulls down the energy levels of valence electrons and increases the delocalization of their wavefunction, which is known as the Stark effect.^[^ ^74^ ^]^ This results in an increased density of the gathered charges, and an enhancement of the BIEF. A thicker depleted layer means an enlarged modulating area of the electronic state and increased electron transfer kinetics. Contrarily, under the reversed voltage the BIEF becomes weaker and the space charge region becomes narrower. When the positive bias is applied at the metal side, the rectification effect would break the equilibrium state at the interface of a Schottky junction with metal and *n*‐type semiconductor, and electrons would move from semiconductor to metal, resulting in a decrease in the total potential barrier and an increase in forward current (Figure 2c). In the case of a Schottky junction with metal and *p*‐type semiconductor, when the work function of the metal is smaller than that of the semiconductor, the electrons would gather at the semiconductor side as a result of a high resistance blocking layer generated, in contrast to *n*‐type semiconductor. On the contrary, applied positive bias at the metal side would stimulate the electrons transfer from the *p*‐semiconductor to the metal, so that additional energy to overcome the potential barrier at the depletion region would be required, which results in inferior conductivity. Accordingly, applied negative bias would decrease the potential barrier, and improve the conductivity of a *p*‐type semiconductor.

Schottky junctions which possess an atomically sharp interface with a high density of states can improve the poor conductivity of transition metal compounds, especially for metal oxides/hydroxides/oxyhydroxide, which show strong localization of electrons owing to the high electrophilicity of oxygen.^[^ ^78^ ^]^ For example, FeOOH/Co/FeOOH heterostructure was used to improve the poor conductivity (10^−5^ S cm^−1^) of FeOOH, and less charge accumulation has been found around FeOOH in contact with Co.^[^ ^79^ ^]^ Other examples, such as NiFe LDH/NiTe,^[^ ^80^ ^]^ MoN@NiFe‐LDH,^[^ ^81^ ^]^ and Cu‐NiCo LDH/NiCo (LDH being layered double hydroxide) heterostructures,^[^ ^82^ ^]^ also proved the Stark effect in Schottky junctions. However, under negative bias, the number of electrons transferring from semiconductor to metal would decline as a result of the increased negative charges at the metal side, which would amplify the potential barrier at the semiconductor side and render a lower reverse current.

### 3.2 *p‐n* Junctions

When *p*‐type and *n*‐type semiconductors are in tight contact, holes and electrons, driven by concentration polarization, would spontaneously flow across the interface toward the counterpart. Simultaneously, the BIEF would be formed to hinder the further transfer of the charge carriers, and the edges of CB and VB would bend upward for *n*‐semiconductor and downward for *p*‐semiconductor until their Fermi levels reach the same level. Eventually, electrons and holes would gather at the side of the *p*‐type and *n*‐type semiconductor, respectively, so that the electron configuration becomes modified at the space charge region.^[^ ^83^ ^]^ This configuration is flexible enough to obtain modulated adsorption energies for intermediates binding at the interface by choosing suitable *p* and *n* semiconductor to construct the junction.

Similar to Schottky junctions, *p*‐*n* junctions also show a rectification effect: when the *p*‐type side is under an external positive bias, the intrinsic potential barrier of BIEF for the drift of electrons decreases, which further promotes the electron transfer toward the *p*‐semiconductor (Figure 2d). Conversely, under negative bias potential, this barrier would prevent the drift of electrons, leading to the absence of the current flow. Li and coworkers^[^ ^76^ ^]^ reported how the rectification effect occurring between *n*‐type metal chalcogenides and *p*‐type iron phthalocyanine (FePc) influenced the ORR performance. They employed conductive atomic force microscopy to measure the current‐voltage curves of FePc on a GaS support. The junction current was only observed at the positive bias surpassing 0.3 V, whereas bare GaS did not respond in a similar manner (Figure 2e). This rectification suggested that electron transferred from GaS to FePc, increasing the number of unpaired *d* electrons at the Fe site. Notably, the Fe center in FePc lay directly above the S atom, where their strong interaction induced upward tilting of the four isoindole units. The accumulation of electrons and disrupted D_4h_ symmetry of structure promoted a spin transition at the Fe center from an intermediate state to a high spin state. This transition resulted in more single‐occupation, π‐symmetry *d* orbitals available for the O_2_ adsorption and dissociation. Similarly, when FePc was placed onto *n*‐type semiconductors: the 2H‐MoS_2_, 2H‐MoSe_2_, 2H‐WS_2,_ or GaS supports with sequentially incremental work functions of −5.07, −4.57, −4.73, and −4.49 eV, a nearly linear rise of the relevant kinetic current densities at 0.85 V versus RHE was detected for the ORR process. However, when *p*‐type GaS with an even higher work function (−0.44 eV) was used as a support, its activity dramatically deteriorated. In such a *p*‐*p* junction, the concentration difference stimulated the diffusion and accumulation of holes around the Fe‐N_4_ moiety of FePc, which, opposite to that of the *p*‐*n* junctions, decreased the unpaired *d* electrons and thus diminished the intrinsic ORR activity.

### 3.3 *p‐p* and *n‐n* Junctions

As discussed above for the *p‐n* junction, the polarization interaction at the interface can achieve simultaneous exposure of electron and hole rich regions, which can facilitate both reduction and oxidation reactions. This scenario can also happen for the *p‐p* and *n‐n* junctions. Due to the presence of holes as the majority of charge carriers, *p*‐type semiconductors are capable of trapping electrons from intermediates, which accelerates the oxidation reaction. Thus, the formation of *p‐p* junctions would further enhance the oxidation ability as a result of accumulated holes. Similarly, *n‐n* type junctions with accumulated electrons demonstrate an enhanced reduction activity by donating electrons to reactants, once the electron density of state becomes adjusted near the Fermi level. Hexagonal boron nitride (*h*‐BN) is regarded as a potential electrocatalyst for nitrogen reduction reaction in view of its weak hydrogen adsorption. To overcome its electron‐deficient structure, Du and coworkers^[^ ^77^ ^]^ realized the *n‐n* junction, composed of *h*‐BN and Cu_3_(HITP)_2_ (HITP = 2,3,6,7,10,11‐hexaiminotriphenylene), where the electrons were able to flow from *h*‐BN (work function = 3.7 eV) to Cu_3_(HITP)_2_ (work function = 4.1 eV) (Figure 2f). The calculated projected density of states (DOS) of the Cu–N(B) active center showed a lower electron density compared to absorbed N_2_ molecules, which confirmed the effective local electron transfer from this active site to the latter, driven by the electron accumulation on Cu sites adjacent to N and B, and resulting in an enhanced N_2_ reduction ability.

Similarly, if a positive potential bias is applied at the side with a lower concentration of holes, the potential barrier would inhibit the transfer of holes, leading to the absence of current flow at a certain range of potential due to the rectification effect. But if negative potential bias is applied, the electrons would pair with holes and subsequently promote the hole transfer from the high concentration side. In this case, the heterostructure allows for the flow of holes across the interface, thus enhancing the oxidation ability. At the same time, when a negative potential bias is applied at the side with a higher concentration of electrons, the heterojunction would also promote the electrons transfer across the interface. Based on this consideration, *p‐p* junctions such as NiSe_2_/FeSe_2_,^[^ ^67^ ^]^ or CoP‐CoO,^[^ ^68^ ^]^ and *n‐n* junctions such as Co_2_N_0.67_‐BHPC^[^ ^84^ ^]^ or CoFe‐LDH@NiCoP/NF,^[^ ^85^ ^]^ were used as catalysts for OER, and ORR as well as HER, respectively.

### 3.4 Metal–Metal Junctions

In metal–metal junctions, the work function gap would require electrons to flow across the heterointerface until their Fermi energies are tuned to the same level. Similar to other junctions, it is conducive to modulate the valence electronic state of active phases and the adsorption states of reactants, intermediates, and products. Importantly, the active metal phase with a high work function can be stabilized through tight contact with the metal with a low work function, according to the “*cathodic protection*” mechanism. As an example, metallic Ir was considered as an alternative hydrogen oxidation electrocatalyst in alkaline exchange membrane fuel cells on account of the appropriate adsorption of hydroxyl.^[^ ^86^ ^]^ However, highly oxophilic Ir can easily be oxidized in this process and experiences higher affinity with hydroxyl. Epitaxially grown Ir nanoclusters on Pd nanosheets could continuously operate at 0.1 V versus RHE for 10 h with negligible current density decay (<6.7%), whereas Ir/C catalyst experienced ≈60% current density loss under the same condition. Electron transfer from Pd to Ir, reflected in the positive shift of Pd K‐edge adsorption spectra, inhibited Ir species from being totally oxidized and thus improved its stability as a hydrogen oxidation electrocatalyst.^[^ ^87^ ^]^ Moreover, BIEF does not happen in metal–metal junctions as a result of their overlap of the conduction band and valence band. This kind of junction commonly shows the same potential as the applied bias, meaning that the rectification effect is hardly to occur.

## 4 Heterostructure Effects

The properties of atoms located at the interface differ a lot from those in the bulk, much like the difference between the turbulent surface and the steady abyssal sea. Electron configuration, geometric structure, and the microenvironment of the catalyst's surface together determine the reactive performance. The former can be modulated by the ligand effect and space charge region, whereas the effective range of the latter can be 100 fold that for the ligand effect. Similarly, the lattice strain is capable of modifying the electron states over a longer range, similar to the space charge region, but it offers more flexibility via control of the thickness and curvature of the substrate, rather than just selecting specific elements in a heterostructure. Moreover, the BIEF as a product of the separate accumulation of electrons and holes has a strong interaction with the microenvironment of the electric double layer at the interface of the electrode. The architecture of heterostructure can also improve the electrochemical performance by accelerating the kinetic process. These are factors inherited in heterostructured electrocatalysts which we will consider in this section.

### 4.1 Space Charge Region

**Figure 3:**
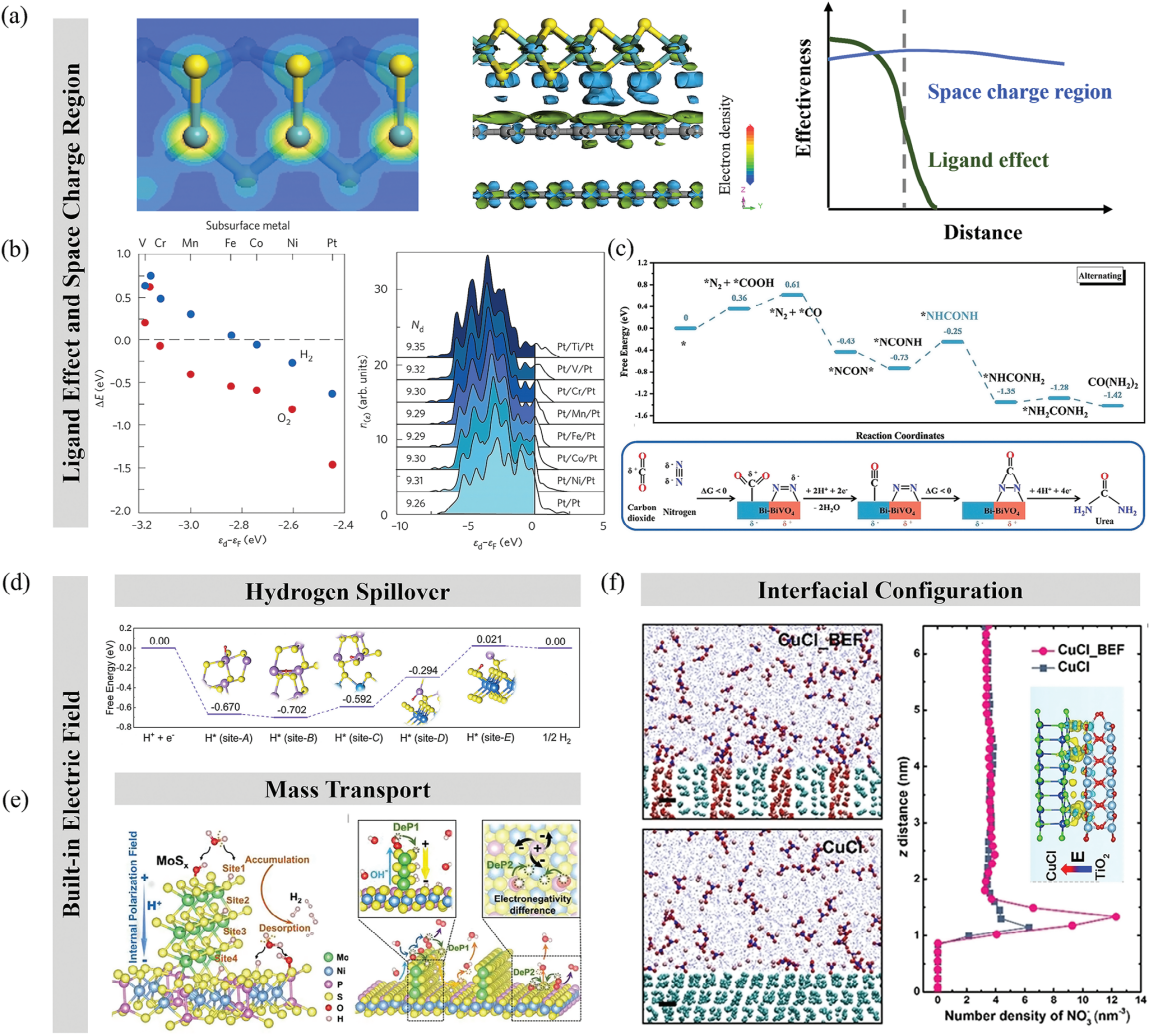
a) Differential charge density of trigonal MoS_2_, van der Waals heterostructure of MoS_2_ and graphene, and the effective length relative to ligand effect and space charge region. b) Adsorption energies of H_2_ and O_2_ versus *d* band center on different Pt‐M‐Pt sandwich structures, and the effect of sandwiching a guest metal layer as the first subsurface layer under Pt surface. Reproduced with permission.^[^ ^89^ ^]^ Copyright 2004, American Institute of Physics. c) Mechanism of the electrocatalytic urea synthesis employing the Bi‐BiVO_4_ Mott‐Schottky heterostructure.^[^ ^90^ ^]^ Copyright 2021, Wiley‐VCH. d) Schematic illustration of the hydrogen spillover for RuS_x_/NbS_2_ electrocatalyst owing to strong BIEF.^[^ ^91^ ^]^ Reproduced with permission. Copyright 2024, Wiley‐VCH. e) Illustration of dual deprotonation enhanced OER for the MoS_2_/NiPS_3_ system based on internal polarization field. Reproduced with permission.^[^ ^92^ ^]^ Copyright 2022, Wiley‐VCH. f) Distribution of anions along the z‐axis electrode distance for CuCl_BEF and CuCl in KNO_3_ solution based on molecular dynamics simulation. Reproduced with permission.^[^ ^93^ ^]^ Copyright 2021, Wiley‐VCH.

Alloying, doping, and forming a compound are commonly used strategies to improve the activity of a catalyst through the *ligand effect* of the coordination element of active sites. Atomic orbitals hybridize and split into bonding and antibonding molecular orbitals located near respective atomic orbitals, and subsequently valence electrons of an atom with weaker electronegativity partially transfer toward the atom with stronger electrophilicity. For example, the differential charge density of MoS_2_ indicated partial electron transfer from the Mo site to the adjacent S site because of the lower energy of the bonding states composed mostly of the atomic states of S, as seen in **Figure** **3**a. In this sense, the adsorption energy of adsorbates can be regulated through the rearrangement of valence electrons of the active site by varying its coordinated atom.^[^ ^88^ ^]^

However, the effective range for the ligand effect is no more than 3 atomic layers. For example, the ligand effects for a Pt layer with heteroatoms in the sublayer were more pronounced when the solute atoms were in the second layer, and became negligible for solute atoms embedded in the fourth atomic layer.^[^ ^94^ ^]^ However, this becomes more complicated when heterostructures with certain thicknesses are formed, on account of the so‐called long‐range space charge region. As considered in Section 3, a similar charge redistribution near the interface in an energy band scale would occur spontaneously due to the work function gap and disparate carrier concentration until the Fermi levels reach equilibrium, as a result of the generation of space charge region.^[^ ^95^ ^]^ While this process is similar to the ligand effect, the distance range for the space charge region is about one order of magnitude larger than that for the ligand effect; it could easily stretch up to several nanometers and usually depends on the Debye length.^[^ ^96^ ^]^ Thus, the construction of heterostructures can be beneficial for modulating the electron state within a longer range as compared to alloying, doping, and forming compounds.

For transition metal‐based electrocatalysts, the binding energy of adsorbate at the surface includes the contribution from the coupling of the adsorbate states and the metal states, namely free‐electron‐like *s*‐electrons and transition metal *d* electrons. Since *s* bands of transition metals are broad and always half‐filled, the interactions between the adsorbate state and *s* electrons of different metals are assumed to be similar. The *d* band state of a metal is taken into account as the key part affecting the binding energy of adsorbates, which depends on the energy of the adsorbate state (*ɛ_a_*), the density of states for the *d* band projected onto the metal atoms in direct contact to the adsorbate (*ɛ_d_*), and the coupling Hamiltonian matrix element between the adsorbate and metal. When inserting the heterometal layer into the metal subsurface, *ɛ_a_* and the matrix element remain almost unchanged, and only the density of states varies. For this heterostructure, *d* band filling changes of surface metal are reflected by the shift of the *d* band center toward the Fermi energy, which is the median value of the integration of the projected surface *d* band density of states up to Fermi level.^[^ ^97^ ^]^ For instance, the *d* band for Pt in the near‐surface of Pt‐M‐Pt (M = Ti, V, …, Co, Ni) is lower in energy and broader in width than for pure Pt slab (Figure 3b), and the shift magnitude of the *d* band center for Pt increases as the subsurface metal M moves to the left of the periodic table.^[^ ^89^ ^]^ In order to eliminate the effect of the lattice strain and synergistic effects of the Cu layer in the subsurface of the Pt lattice, a study by Stephens et al. was designed to solute Cu under the Pt plane (Cu/Pt (111)) for OER. Assuming that this heterostructure was unstrained, through DFT calculations, they found out that with an increased amount of Cu in the second layer of Pt lattice, Gibbs free energy of *OH on Pt showed a linear scaling in a least‐square fit. When the Cu coverage reached 1/2, Cu/Pt (111) showed the most modulated adsorption energy of *OH, and the voltammograms represented an 8‐fold increase of activity as compared to pure Pt (111) at 0.9 V (vs RHE).^[^ ^98^ ^]^

Importantly, the asymmetric charge distribution at the space charge region significantly enhances the characteristic electrocatalytic activities of the heterostructure.^[^ ^99^ ^]^ For example, the coexistence of electrons and holes accumulation region in heterostructures of MoSe_2_‐Cu_2_S,^[^ ^100^ ^]^ CoPc‐CH_3_/CoS,^[^ ^101^ ^]^ and CoNiP@NiFe LDHs,^[^ ^102^ ^]^ enhanced their respective oxidation and reduction performance as bifunctional electrocatalysts for water electrolysis. Advanced heterostructures were also designed as an integration of nanoreactors, parallel and in‐series, to produce complex organic products.^[^ ^103^ ^]^ Heterostructures exhibiting both electrophilicity and nucleophilicity were explored as potential electrocatalysts for urea synthesis. Yuan et al.^[^ ^90^ ^]^ reported a Mott‐Schottky type Bi‐BiVO_4_ heterostructures capable of electrochemically converting CO_2_ and N_2_ molecules into urea. The C–N coupling occurred at the space‐charge region of the heterointerface, and promoted adsorption and activation of inert N_2_ and CO_2_ molecules at electrophilic Bi and nucleophilic BiVO_4_ regions, as illustrated in Figure 3c. Compared to the pristine BiVO_4_, Bi‐BiVO_4_ heterostructure suppressed the competitive conversion of *N_2_ into *NNH and alleviated the CO poisoning; this facilitated the formation of *NCON*, a key intermediate that determined the selectivity of the electrocatalytic urea production. As a result, the Bi‐BiVO_4_ heterostructure achieved a remarkable urea yield rate of 5.91 mmol h^−1^ g^−1^ and a Faradaic efficiency of 12.55 % at −0.4 V versus RHE. Similarly, nitrate was also used as a nitrogen source to replace N_2_ and participated in the cascade reaction with CO_2_ to produce urea. Wei et al.^[^ ^104^ ^]^ found out that Ag‐CuNi(OH)_2_ heterointerface facilitated the coupling of *CO on silver reduced from CO_2_ and *NH_2_ on a hydroxide reduced from nitrate ions, resulting in a high urea yield rate of 25.6 mmol g^−1^ h^−1^ and a Faradaic efficiency of 46.1 %.

### 4.2 Built‐In Electric Field Effects

The strength of BIEF is a representative descriptor to quantify the state of the depletion layer. The potential of BIEF (*ΔU*) can be calculated as *ΔU = ΔΦ/e*, where *ΔΦ* is the work function difference of two phases of a heterostructure, and *e* is the electron charge. Then, the strength of BIEF can be calculated as: *E = ΔU/d*, where *d* is the thickness of the heterostructure. Experimentally, the strength of BIEF can be determined from the surface potential (V_s_) and the surface charge density (*ρ*), which can be measured by Kelvin probe force microscopy or UV photoelectron spectroscopy, and calculated using Zeta potential. Specifically, *E* can be obtained by the following formulae:^[^ ^105^ ^]^

(8) $$E={\left(\frac{2V_{s}\rho }{\varepsilon \varepsilon_{0}}\right)}^{\frac{1}{2}}$$

(9) $$\rho =\sqrt{8cN_{A}\varepsilon_{0}\varepsilon k_{B}T}\;\sinh\frac{e\zeta }{2k_{B}T}$$

where ε is the low‐frequency dielectric constant; ε *_0_* is the vacuum dielectric constant; *c* is ion concentration; and *ζ* is Zeta potential.

Other than modulating the electron configuration, BIEF can adjust the microenvironment of the interface, where the reactions occur. Liu and coworkers^[^ ^106^ ^]^ emphasized the importance of hydrogen spillover under high potential to accelerate the HER kinetic. When the applied potential becomes more negative, hydrogen spillover from Ru to the CoP surface occurs. Due to the nearly‐zero Gibbs hydrogen free energy on CoP, the entire alkaline HER process becomes significantly accelerated, resulting in superior activity at the ampere‐level current densities (357 mV at 1 A cm^−2^). Yue et al.^[^ ^91^ ^]^ designed RuS_x_/NbS_2_ heterostructure exhibiting hydrogen spillover effect, which was attributed to the nonuniform density of states at the active sites of the interface. As seen in Figure 3d, Gibbs hydrogen free energy was found to gradually increase from negative value to zero when the adsorbed Ru site was located close to the interface. This proximity creates a forming channel for the hydrogen to spillover to the S site on the basal plane of NbS_2_. The color of the mixture of RuS_x_/NbS_2_ and WO_3_ changed from yellow to dark‐blue after HER occurred, since the spilled‐over hydrogen reacted with WO_3_ to form dark‐blue colored H_x_WO_3_. In contrast, no color change was observed for individual RuS_x_/NbS_2_ and WO_3_ samples.

Apart from accelerating the hydrogen spillover, the internal polarization field in the MoS_2_/NiPS_3_ heterostructure facilitated hydroxyl diffusion and enabled MoS_2_‐to‐NiPS_3_/P‐to‐S dual‐pathways for the deprotonation processes *OH → *O and *OOH → O_2_ (Figure 3e).^[^ ^92^ ^]^ On the other hand, BIEF can increase local reactant concentration and thus accelerate the reaction kinetics.^[^ ^107^ ^]^ Lu and coworkers^[^ ^93^ ^]^ utilized molecular dynamics simulation and proved that the CuCl_BEF (heterostructure comprising CuCl and TiO_2_ components) attracted a higher concentration of K^+^ and ${\mathrm{NO}}_{3}^{-}$ ions at the interface with electrolyte compared to pure CuCl during the nitrate electroreduction process (Figure 3f).

Electrochemical measurements showed that both hydrogen adsorption and hydrogen evolution processes are sluggish in an alkaline medium, which is in line with the observation of rate‐determining step change for HER caused by the deficiency of the available protons.^[^ ^108^ ^]^ Thus, the rate of this charge transfer step is influenced by the interfacial water network in the Helmholtz layer that can accommodate proton migration. As long as the water network is soft and easily reoriented, the proton transfer through the double electric layer would be accelerated. On the other hand, if the network is rigid and hard to adjust, the proton transfer would be decelerated.^[^ ^109^ ^]^ At the HER potential, the interfacial water network interacts strongly with the interfacial electric field, which is influenced by the charge distribution between the electrode and electrolyte. Close to the potential of zero charge, interfacial water molecules are relatively free to adjust for easily proton transfer, while under a strong electric field, which is further away from the potential of zero charge, water molecules become more rigid and difficult for reorientation. Koper and coworkers^[^ ^110^ ^]^ revealed that decorating Ni(OH)_2_ on the Pt surface resulted in a negative shift of zero charge potential, which lowered the interfacial electric field and thus decreased the energetic barrier for the reorganization of the interfacial water network. Their study also offered an interpretation of the decreased thermal coefficient of the double layer potential based on the slope of potential decay after a laser‐induced temperature‐jump. However, the proton transport via the hydrogen bond network outside the inner Helmholtz layer can be accelerated owing to the highly ordered network with oriented induced dipole induced by the electric field.^[^ ^111^ ^]^

### 4.3 Synergistic Effects

Components of a heterostructure can provide different adsorption energies of intermediates and thus enable more favorable reaction pathways due to the interplay at the interface.^[^ ^112^ ^]^ Here, we will consider the electrochemical OER process as an example to discuss how the architecture structure of heterointerface can help to break the scaling relation limitation. Under the adsorbate evolution mechanism (AEM), the LSR of the binding energies among the intermediates, *OH and *OOH, which is attributed to their comparable HOMO levels, inevitably results in the intrinsic overpotential in the range of 0.27–0.47 V.

**Figure 4:**
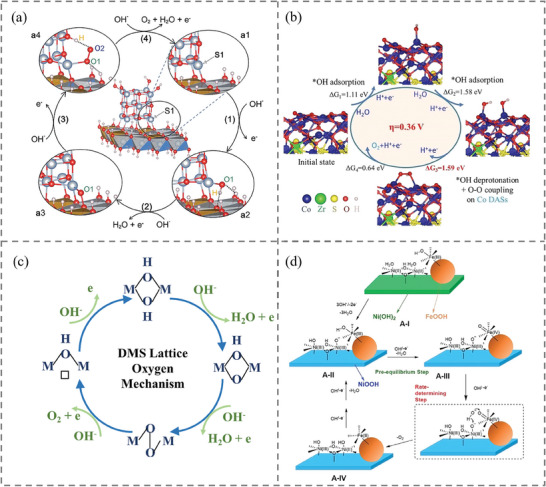
Strategies for breaking the limitation of scaling relation for OER process via synergistic interaction of heterostructured catalysts: a) Schematic illustration of the dynamic 3D adsorption of oxygenates within OER pathway at the interface of NiO/NiFe LDH. Reproduced with permission.^[^ ^114^ ^]^ Copyright 2019, Wiley‐VCH. b) Adsorbate evolution mechanism following the O─O coupling mechanism based on the suitable Co–Co distance at the intersection of Zr‐doped Co_9_S_8_/Co_3_O_4_ heterostructure. Reproduced with permission.^[^ ^116^ ^]^ Copyright 2023, Wiley‐VCH. c) Schematic of a dual‐metal‐site lattice oxygen mechanism for oxygen reduction reaction; d) Schematics of the bifunctional AEM and LOM coupling pathways for γ‐FeOOH‐NiOOH. Reproduced with permission.^[^ ^117^ ^]^ Copyright 2020, Wiley‐VCH.

As we discussed above, adsorption energies of *OH and *OOH intermediates depend on the Hamiltonian matrix element between the adsorbate and the active site. On this basis, several strategies aiming at breaking the limitation of scaling relation for the OER process via synergistic interaction of heterostructured catalysts have been reported.^[^ ^113^ ^]^ A dynamic 3D adsorption model for *O, *OH, and *OOH intermediates at the intersection of NiO and NiFe LDH was proposed by Du and coworkers, who explained how the disparity in Gibbs free energies for *OH and *OOH at the interfacial Ni^4+^ site (denoted as S1 in **Figure** **4**a) decreased from the theoretical value of 3.2 to 2.75 eV.^[^ ^114^ ^]^ As seen in Figure 4a, the alternative adsorption of oxygen‐containing intermediates occurs at the interface, involving the O and Ni sites onto the NiO cluster, as well as the O site within NiFe LDH, with the assistance of hydrogen bonds. By following the two‐electron pathway to form the adsorbed O intermediate and accelerating the formation of oxo‐oxo coupling, the scaling relation can be broken by avoiding the generation of *OOH. Inspired by CaMn_4_O_5_ binuclear catalyst,^[^ ^115^ ^]^ the photosystem II in nature's oxygen‐evolving complex, Wang et al.^[^ ^116^ ^]^ considered the OER process that involved binuclear active sites and oxo‐oxo coupling mechanism. They realized Co_9_S_8_/Co_3_O_4_ heterostructure containing atomically dispersed zirconium with moderate interatomic Co–Co distance (ca. 2.80 Å) at the interface (Figure 4b). In situ synchrotron radiation Fourier transform infrared micro‐spectroscopy revealed the presence of a characteristic peak at 1090 cm^−1^ attributed to the oxygen bridge formation, indicating that the O─O coupling OER process occurred at adjacent Co─Co sites. In contrast, when *OOH intermediate adsorbing on the Co site was involved in the case of the bare Co_9_S_8_, another characteristic peak was detected at 1024 cm^−1^ and the O─O peak at 1090 cm^−1^ disappeared. Electrochemical tests demonstrated that the zirconium‐doped Co_9_S_8_/Co_3_O_4_ only required an overpotential as low as 155 mV, whereas Co_9_S_8_ needed a value of 230 mV to deliver 10 mA cm^−2^.

Nickel‐iron compounds, and especially NiFe LDHs with brucite‐like structures, are recognized as high‐performance OER catalysts whose performance is comparable with IrO_2_ and RuO_2_ under alkaline electrolysis. Dual‐metal‐site lattice oxygen mechanism (DMS LOM, which is illustrated in Figure 4c) was responsible for these surface oxygen‐rich electrocatalysts, which follows a direct coupling of two adjacent *O with the participating of lattice oxygen and avoids the formation of *OOH intermediate species.^[^ ^118^ ^]^ However, dynamics that do not support the O─O coupling may restrain the improvement of OER kinetics. Hu and coworkers^[^ ^117^ ^]^ reported γ‐FeOOH‐NiOOH heterostructure which was significantly more active than NiFe LDH. Isotope exchange experiments (^16^O/^18^O and H/D) were used to analyze the OER mechanism. While 16% lattice oxygen exchange occurred in NiOOH, there was no such exchange observed in γ‐FeOOH, which indicated that the LOM mechanism occurred in NiOOH. Simultaneously, a lower H/D isotope effect (k_H_/k_D_ = 1.4 – 2) indicated a kinetic isotope effect in proton/hydrogen transfer rather than a thermodynamic one after D‐substitution in γ‐FeOOH‐NiOOH, thus pointing out AEM mechanism occurring in γ‐FeOOH. Based on these results, a bifunctional mechanism was proposed to explain the 10 times increase in the turnover frequency, which is illustrated in Figure 4d. At the interface the Fe^III^ center in γ‐FeOOH first underwent an AEM mechanism to form an electrophilic Fe^IV^ = O center, and subsequently, the Ni^III^‐O moiety captured an external OH^−^ and the Fe^IV^ = O reacted with O in a fixed OH^−^, leading to desorption of O_2_. A similar sequential mechanism was also suggested by Xin et al.^[^ ^119^ ^]^ for the heterostructure based on Fe─Co(OH)_2_/Fe_2_O_3_. Here, Fe─Co(OH)_2_ facilitated the rapid deprotonation following the AEM process to produce adsorbed *O, while Fe_2_O_3_ underwent the LOM mechanism to trigger the O─O coupling step. As a result, this electrocatalyst only required an overpotential of 249 mV to deliver a current density of 100 mA cm^−2^.

Furthermore, it was reported that heterostructures composed of the reactive side as well as proton or electron‐donating side, such as polypyrrole, polyaniline, and polyindol, are capable of breaking the LSR limit by influencing the adsorption of the intermediate oxygen species on the active sites through modifying the catalytic microenvironment.^[^ ^120^ ^]^ Song and coworkers^[^ ^121^ ^]^ found out that proton transfer occurred from the secondary amine hydrogen in protonated polypyrrole to specific oxygen surface intermediates on transition metal terephthalates (MTP, M = Fe, Co, Ni, Cu, or Zn) with the assistance of hydrogen bond. The proton transfer involved different kinds of intermediates across those MTPs, namely *OO for CuTP, *OOH for FeTP, and *O for CoTP, NiTP, CuTP, and ZnTP. Taking CoTP as an example, the free energy barrier between the two intermediates (*OO and *OOH) in the presence of protonated polypyrrole decreased to +0.61 eV, compared with deprotonated polypyrrole (+0.99 eV), which thus broke the LSR limitation within OER.

### 4.4 Lattice Strain Effects

**Figure 5:**
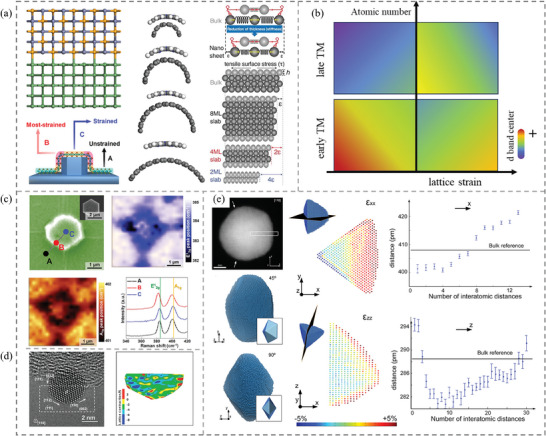
a) Lattice strain is induced by the lattice mismatch of the heterostructure, as well as the curvature and thickness of the constituting components. Reproduced with permission.^[^ ^123^ ^]^ Copyright 2019, American Association for the Advancement of Science. b) Shift of the *d* band center of the early and late transition metals under tensile and compressive strain. c) Confocal Raman measurements provide a mapping of the strain distribution on the 1L‐MoS_2_/ZnO heterostructure. Reproduced with permission.^[^ ^124^ ^]^ Copyright 2019, American Chemical Society. d) Aberration‐corrected HRTEM image of de‐alloyed Pt‐Fe nanoparticle and the mappings of the lattice strain relative to the bulk Pt lattice. Reproduced with permission.^[^ ^125^ ^]^ Copyright 2012, American Chemical Society. e) HAADF‐STEM projection image of an Au nanodecahedron, 3D visualization of its reconstructed nanoparticle, and 3D strain analysis of the slice through ε_xx_ and ε_zz_ volume. Reproduced with permission.^[^ ^126^ ^]^ Copyright 2015, American Chemical Society.

Lattice strain, also denoted as a geometrical strain, is an important characteristic in the field of heterostructured catalysts. Generally, lattice strain in heterostructures arises from the lattice mismatch and the difference in the atomic coordination because of the imparity of composition and occurrence of some defects at the interface and includes compression and tension on the two counterparts simultaneously. Besides, the thickness and curvature of the constituting components can also affect the distribution of the interfacial lattice strain (**Figure** **5**a).^[^ ^122^ ^]^ An ideal average lattice strain for a heterostructure can be calculated using the equation, *S* = $\frac{\left(\alpha_{I1}-\;\alpha_{I2}\right)}{\alpha_{I2}}$, where α_*I*1_ and α_*I*2_ represent the lattice constant of the two components, respectively.

Lattice force, similar to the ligand effect, is capable of modulating the local electronic state, by shifting the *d* band center of transition metals toward or away from the Fermi level, which is related to the binding strength of the adsorbates.^[^ ^127^ ^]^ Importantly, the shift of the *d* band center is aligned with the magnitude of lattice strain, which is much easier to control and measure. Unlike in the case of the limited effective atomic distance involved in both the ligand effect and synergetic effect, the impact of lattice strain extends over larger distances, even in a Van der Waals heterostructure.^[^ ^128^ ^]^ Generally, lattice strain is uniform within a thin film of a few monolayer thicknesses due to atomic relaxation, while the developed stress becomes non‐uniform and notably decreases when the film thickness increases. As an example, it was reported that the lattice strain of GeTe grown on a Bi_2_Te_3_ layer of ≈4 nm thickness decayed exponentially with increasing thickness, while this effect vanished when the GeTe film reached a thickness of 25 nm.^[^ ^129^ ^]^

The strain effect on *d* band electrons for late and early transition metals, whose number of *d* electrons is more than 5 and less than/equal to 5, respectively, operates in an opposite way (Figure 5b). For the former, the expansion of interatomic spacing introduces a less overlap of the *d* orbitals and a narrowed *d* band width. In this case, the upshift of *d* band center enhances the interaction between the adsorbate and the surface. Accordingly, when the material is under compressive strain, weaker binding occurs because of the downward shift of the *d* band center.^[^ ^130^ ^]^ Taking Pt as an example, 1% tensile (compressive) strain can upshift (downshift) the 5*d* band center by ≈0.1 eV, which dramatically influences the bonding strength and surface reactivity.^[^ ^131^ ^]^ However, early transition metals show weaker adsorption upon lattice expansion.^[^ ^132^ ^]^ This is because fewer electrons occupying the *d* band result in the downshift of the *d* band center, as illustrated in Figure 5b. Additionally, some studies pointed out that a tensile strain of ≈1.5% at the crystal surface can elevate the dissociation constant of the adsorbates by more than three orders of magnitude.^[^ ^133^ ^]^

Different from the ligand effect which requires certain ligand atoms to modify the electronic state of the active site by either attracting or donating electrons, the lattice strain endows modulated electronic states via changing the interatomic distance regardless of the types of ligand atom.^[^ ^128^ ^]^ Obviously, it is also able to modify the geometric structure, thus allowing us to adjust the coordination environment and the adsorption energies of active sites. An example was reported by Ye and coworkers,^[^ ^122^ ^]^ who deposited cobalt phthalocyanine (CoPc) onto carbon nanotubes with diameters ranging from 1 to 100 nm. The curvature of CoPc upon these nanotubes varied in the abroad range of 96° to 1.5°, which resulted in high selectivity of the production of methanol rather than CO during electrochemical CO_2_ reduction. X‐ray absorption near‐edge structure and extended X‐ray absorption fine structure analysis showed that when the carbon nanotube's diameter decreased, both the Co–N1 (first coordination sphere) and the Co–C1 (second coordination sphere) distances increased. In addition, the intensity of the Co–C1 peak increased due to strong Co–C interactions. DFT analysis showed that the curved CoPc experienced stronger CO binding than the flat CoPc, which was also supported by experimental data, as the yield ratio of methanol increased from 13.9% to 53.2%. For the flat CoPc, generated CO experienced immediate desorption, making further formation of methanol unlikely, while for the curved CoPc, CO remained bound to the catalyst's surface, which enabled its further transformation to methanol.

The strain effect on the interactions between the adsorbate and surface is rather complex: adsorbates can either push neighboring atoms outward or pull them inward, thus inducing a compression or tensile stress in the adjacent area. Peterson and coauthors^[^ ^134^ ^]^ found out that when CH_2_ was attached at the bridge site of biaxially strained Cu [110], the neighboring metal atoms were pushed outward, while, when CH_2_ was adsorbed at the 4 fold hollow sites of Cu [110], they were pulled inward. This strain can further weaken the connection of active moieties, such as IrO_6_ octahedra units in IrO_2_, and thus diminish the structural stability of heterostructure. When the average lattice strain exceeded 5%, the interface of a heterostructure became incoherent and even disassemble.^[^ ^135^ ^]^ For instance, the AuCu@Pt core‐shell structure with a thin Pt layer showed 32% ORR activity loss after 30 000 cycles, which was worse than for a thick Pt layer (10% loss). This discrepancy was attributed to the greater compressive strain imposed on the thinner Pt shell by the AuCu core, which has a smaller lattice parameter.^[^ ^136^ ^]^ However, this strain could be relieved by applying an inverse stress onto the active phase in a heterostructure.^[^ ^137^ ^]^ Iridium oxides, as state‐of‐the‐art OER electrocatalysts, often suffer degradation under electrolysis, as the nucleophilic attack of hydroxyl and the desorption of O_2_ through O─O coupling at the Ir site can weaken the connection between IrO_6_ octahedra units.^[^ ^138^ ^]^ Hrbek et al.^[^ ^139^ ^]^ found out that forming an Ir‐Ru@IrO_x_ core‐shell structure could stabilize the outer amorphous IrO_x_ shell. *Operando* wide‐angle X‐ray scattering analysis indicated that the lattice parameter of the inner metallic Ir‐Ru core remained almost constant under the OER process, whereas the Ir‐Ru/IrO_x_ without the core‐shell structure experienced a rapid expansion of lattice. The compression strain on the IrO_x_ shell imposed by the Ir‐Ru core prevented the degradation, while in contrast, strain loss caused by Ru leaching resulted in poor stability for Ir‐Ru/IrO_x_, though these two kinds of structures showed similar performance in proton exchange membrane water electrolyzer.

To experimentally map the lattice strain, several physical characterization techniques, such as X‐ray diffraction (XRD), Raman spectroscopy, high‐resolution transmission electron microscopy (HRTEM), as well as synchrotron imaging, have been employed, as we will shortly consider below.^[^ ^140^ ^]^

*X‐ray Diffraction*: According to the Bragg formula, the downshift and upshift of the diffraction angle in the XRD spectrum represent the change of the lattice parameter, indicating tensile and compression strain, respectively. Rietveld refinement of X‐ray diffraction or scattering patterns can be applied to determine the micro‐strain, as well as the degree of defectiveness, such as the presence of grain boundaries, stacking faults, and twins. Microstrain can be quantitatively estimated using the Williamson‐Hall equation: $S=\frac{\beta_{hkl}}{4tan\theta }$, where β_*hkl*_ represents the width at the half maximum of the XRD peak, and θ is half the value of the diffraction angle.^[^ ^141^ ^]^

*Raman Spectroscopy*: The magnitude and the spatial distribution of strain can also be determined by Raman spectroscopy. Red or blue shift of the vibration or stretching peaks, such as E_2g_ and A_1g_ peaks in MoS_2_, or 2D and G peaks in graphene, can be used to monitor the lattice strain, which is estimated as: $S=\frac{\Delta \omega }{\chi }$, where *χ* indicates the shift rate of Raman modes. According to previous reports, the shift rate of the E_2g_ mode in MoS_2_ under biaxial strain is ≈3.5 cm^−1^ per % strain,^[^ ^142^ ^]^ while for 2D and G peaks of graphene, it is ≈27.7 and 14.2 cm^−1^ per % strain in one direction, respectively.^[^ ^143^ ^]^ Confocal Raman measurements were carried out by Zhang et al.^[^ ^124^ ^]^ to obtain the distribution mapping for the E_2g_ and A_1g_ peak frequencies of 1L‐MoS_2_ on hexagonal ZnO nano‐arrays. As seen from the Raman mapping in Figure 5c, the most‐strained region is at the top edge of the ZnO nanorod (point B), the medium‐strained region is at the flat ZnO substrate (point A), and the almost unstrained region is at the top of the ZnO nanorod (point C).

*High‐Resolution Transmission Electron Microscopy*: Geometric phase analysis of HRTEM images, which provides detailed spatial information and the deformation degree of nanomaterials, is a semi‐quantitative lattice image processing method. It involves focusing a small aperture on a region with strong reflection in the Fourier transform of an HRTEM image and then performing an inverse Fourier transform. This method operates in reciprocal space and analyzes the spatial frequency phase shift, in terms of which the resulting phase component of the complex image offers information about local displacements of atomic planes. Thus, strain distribution can be revealed by analyzing the derivative of the displacement field.^[^ ^144^ ^]^ On the other hand, the peak‐finding method is applied in real space, superimposing a 2D reference lattice extrapolated from a non‐distorted region of the material onto the HRTEM image, and calculating the local discrete displacement field at each node.^[^ ^145^ ^]^ Still, there are challenges that need to be overcome before these methods can achieve adequate accuracy and reliability. Computational methods have been developed to extract information from HRTEM images and to eliminate the distortions introduced by the microscope itself.^[^ ^146^ ^]^ Zhu and coauthors^[^ ^125^ ^]^ developed an approach to characterize the lattice strain using an aberration‐corrected HRTEM, in which they established the area of each triangle unit bounded by nearest distance along $\left[\bar{1}12\right]$, $\left[1\bar{1}2\right]$ and $\left[1\bar{1}0\right]$ directions to provide 2D lattice strain mapping (Figure 5d). Moreover, these methods only offer 2D imaging of the lattice strain, which does not represent the actual 3D material. Sara et al.^[^ ^126^ ^]^ applied the electron tomography technique, which is based on a series of 2D projection images acquired at different tilt angles (from −74° to 68°), and combined a 3D Gaussian function to reconstruct a 3D Au nanodecahedron (Figure 5e). They computed derivatives of the displacement map corresponding to ε_*xx*_ and ε_*zz*_ axes, and variation of the lattice parameters along the x and z direction based on the related slices.

*Synchrotron Imaging*: X‐ray imaging is a significantly less damaging technique as compared to HRTEM, which provides 3D mapping as a result of the high penetration capability of X‐rays.^[^ ^147^ ^]^ The advancement of synchrotron radiation sources has greatly promoted X‐ray imaging techniques, as they rely on X‐ray beams with high brilliance, monochromaticity, wide energy range, and coherence. As a result, several powerful techniques, such as X‐ray projection imaging, transmission X‐ray microscopy, scanning transmission X‐ray microscopy, and coherent diffraction imaging (CDI) became available, providing information regarding the spatial displacement of the atoms and lattice strain.^[^ ^148^ ^]^ Imaging resolution of the former three techniques relies on the magnification lens within the detector unit, whereas the phase retrieval of the samples in the CDI approach can be extracted from the interference pattern through consecutive interactive Fourier transforms with an enforced constrain at every iterative step.^[^ ^149^ ^]^ The common iterative algorithms for CDI include error reduction, hybrid input‐output, and relaxed averaged alternating reflectors.^[^ ^150^ ^]^ The CDI technique, especially the scattered intensity around a Bragg reflection (Bragg CDI), offers a high resolution, smaller than the d‐spacing of the considered reflection. It is sensitive to the distortions of the crystalline lattice, because of the proportional function of the phase shift of scattered wave and the vector displacement field in the reconstructed complex density.^[^ ^151^ ^]^ Importantly, this technique can also be applied for 4D mapping of the lattice strain, which includes the variation of the lattice in time, as well as for studying the nucleation and growth of crystals, catalytic mechanisms, etc.^[^ ^150^, ^152^ ^]^ Kim and coworkers^[^ ^153^ ^]^ performed Bragg CDI to monitor the strain dynamics in an individual Pt nanoparticle during catalytic methane oxidation, which demonstrated contraction behavior at the edges and a high‐tensile strain at the surface of the Pt nanoparticle during the oxidation process, where the latter acted as the active region responsible for the highly catalytic performance.

### 4.5 Geometric Effects

The geometry of heterostructures, which includes the atomical configuration of active sites and their macroscopic topological structure, can also accelerate the kinetics of electrochemical processes. As we already discussed above, the local coordination environment of active sites, namely CN and coordination atoms, determines the adsorption state of reactive species. The interfacial geometry of heterostructures, which can be quite different from that of the bulk counterparts, often contributes to high electrochemical performance through a synergistic effect. That's why γ‐FeOOH‐NiOOH showed a tenfold increase of the turnover frequency activity in comparison with NiFe LDHs OER electrocatalyst.^[^ ^5^ ^]^

Reducing the size of one or two components to generate cluster‐cluster or cluster‐support heterostructure on a scale of several nanometer provides more possibilities for exposing the active sites, where the support can be 1D nanorods, 2D nanosheets, or 3D architectures.^[^ ^154^ ^]^ By pyrolysis of N‐doped carbon nanosheets impregnated Ru and Cr ions in the H_2_/Ar atmosphere, and Ru‐CrO_x_ cluster‐cluster heterostructure was fabricated, making use of their different reduction ability.^[^ ^6^ ^]^ This heterostructure exhibited impressive mass activity for hydrogen oxidation reaction; its normalized kinetic current density (13.76 ${A\;\mathrm{mg}}_{\mathrm{Ru}}^{-1}$) and exchange current density (2.8  ${A\;\mathrm{mg}}_{\mathrm{Ru}}^{-1}$) were 14.5 and 3.6 times higher than those of Ru on N‐doped carbon nanosheets. However, stability issues may become the key challenge for these heterostructures, as the clusters tend to ripen into large nanoparticles, dissolute into the electrolyte, and/or strip‐off from the support under harsh reaction conditions. Encapsulating clusters into protective shells, without affecting the migration of reactive species, can improve their stability. Galeano et al.^[^ ^155^ ^]^ found out that Pt nanoparticles located at the outer surface of hollow graphitic spheres almost vanished after 3600 degradation cycles in the ORR potential range, while the density of Pt nanoparticles located inside the pores of those spheres remained at a remarkable extent. Similarly, confining clusters into other kinds of porous materials, such as zeolites, carbon nanotubes, and metal‐organic frameworks (MOF), can also stabilize the whole structure through the confinement effect and inhibit its disassembly under the action of reactive species.^[^ ^156^ ^]^

Importantly, porous supports can also endow the molecular selectivity, so that the transformation of reactants into products would depend on how those molecules fit into the confined space.^[^ ^157^ ^]^ For example, Sargent and coworkers^[^ ^158^ ^]^ encapsulated Ag nanoparticles inside Zr‐based MOFs (Ag/Zr‐fcu‐MOF‐BDC), which separately used 1,4‐benzene dicarboxylic acid (BDC) or its derivates as organic linkers. They found out that grafting the amine functional group (Ag/Zr fcu‐MOF‐NH_2_‐BDC) or changing the organic linker to 1,4 naphthalenedicarboxylic acid (Ag/Zr‐fcu‐MOF‐NDC) could increase the local CO_2_ concentration near Ag nanoparticles by the reduced pore‐aperture sizes. Raman spectra indicated that no CO stretching peak appeared for Ag/Zr‐fcu‐MOF‐BDC. In contrast, a broad peak ≈2100 cm^−1^ indicated an atop adsorption of CO on Ag/Zr fcu‐MOF‐NH_2_BDC, and a narrow peak ≈1950 cm^−1^ demonstrated the bridge mode of CO adsorption on Ag/Zr‐fcu‐MOF‐NDC. As a result of the increased local CO_2_ concentration and less reactive CO at the bridge site, Ag/Zr‐fcu‐MOF‐NDC showed CO Faraday efficiency of 94%, which was higher than Ag/Zr fcu‐MOF‐BDC (74%) and Ag/Zr fcu‐MOF‐NH_2_BDC (80%). Moreover, the selectivity of electrochemical CO_2_ reduction depends on the migration rate of key intermediates. A yolk‐shell nanoreactor Ag@Cu_2_O showed a high CH_4_ Faraday efficiency of ≈74% at −1.2 V versus RHE, in which the moderate CO coverage at Cu_2_O shell was determined by the diffusion rate of CO generated from Ag yolk, which could restrain the formation of hydrogen and C_2+_ products (C_2_H_4_ and C_2_H_5_OH), and promote hydrogenation of CO toward CH_4_.^[^ ^159^ ^]^ Additionally, the limitation of LSR can be broken by the geometry effect. Nanoscopic channels providing a confined reaction environment enabled selective interactions between reaction intermediates and the catalyst to modulate the adsorption state of involved intermediates individually.^[^ ^160^ ^]^

## 5 Conclusion and Perspective

Electrochemical processes offer a great potential to efficiently drive non‐spontaneous reactions and produce oxidation and reduction products at electrodes. It is important to design the most optimized electrocatalysts to reduce costs and enable their large‐scale applications. The microkinetic model sheds light on the conjunction principle of the reaction kinetic rate and the adsorption energy of intermediates. The adsorption energy theory, including the *d* band model and the *p* band model, reveals a significant role of the valence state of the catalyst on the binding energy with adsorbates. When the *d* band center shifts upward, depopulated *d* states result in a strengthened binding; whereas the system with a higher *p* band center results in a weaker binding due to the almost fully occupied antibonding states. In electrochemical processes, the adsorption energy scaling relation among intermediates with similar valence states or HOMO levels causes additional potential barrier. Apart from the valence state, the coordination environment of water molecules within the inner and outer Helmholtz layer also plays an important role in the mass transfer and energy barriers for the adsorption/desorption.

Heterostructured electrocatalysts show great potential to modulate electron states, geometric structure, and microenvironment of the double electric layer. Different types of heterojunctions with varying distributions of charge carriers exhibit specific rectification phenomena in response to applied bias. This can reduce the potential energy barrier for the electron transfer across the interface. In addition to the short‐range ligand effect, heterostructures also show long‐range valence electron redistribution within the space charge area. The induced built‐in electric field can not only accelerate hydrogen spillover and accumulate the counterpart reactant ions at the interface but also redistribute the microenvironment of both the inner and outer Helmholtz layer, relative to the polarization and transport of the water molecules. Simultaneously, synergistic interaction between the two components of the heterostructure can break the limitation of scaling relation through the dynamic evolution of the adsorbing sites at the interface, along with associated mechanism changes. Lattice strain resulting from the mismatch of the interplane distances or the bending of support can also influence and modulate the valence states. Tensile strain enlarges the distance between the atoms of the catalysts and alleviates the overlap of the *d* electron cloud, resulting in the upshift of the *d* band center and a strengthened binding for the late transition metals but weaker binding for the early transition metals. Besides electronic characteristics, the electrocatalysis rate can also be influenced by the geometry of heterostructures which accelerates the kinetic process. Importantly, the confinement effects of the heterostructure can also improve both the stability and selectivity of electrocatalysts.

Even though significant progress has been made in developing the fundamentals of the microkinetic model and the practical realization of the heterostructured electrocatalysts, there are still several challenges that need to be addressed. These include the acquisition of transition state energy, the accuracy of DFT approaches, and a better understanding of the dynamic evolution of heterostructures under operating conditions. Addressing these challenges will require advanced physical characterizations, further refinement of the DFT approaches, and support of artificial intelligence and machine learning to deal with large amounts of data.

*Determination of Transition State Energy*: The microkinetic model and the adsorption energy theory connect kinetics with the valence states of catalysts, making it easier to screen the most optimized catalysts without relying solely on the trial‐and‐error approaches. However, an approximation of the activation energy based on its nearly linear relationship with the reaction energy results in deviations from the real values. This is because it is hard to determine the transition state, especially for multiple‐electron transfer processes, such as 6 electron‐proton coupled nitrogen reduction reaction, which produces several intermediates, such as *N_2_H, *N_2_H_2_, *N_2_H_3_, *N, *NH, and *NH_2_.^[^ ^161^ ^]^ Electrochemical transformations occur instantaneously on the time scale of picoseconds to nanoseconds, making it difficult to detect transition states. In order to study such micro‐structures and short‐living states, considering the typical interatomic distances of ≈1 Å, the incident wave should have a comparable or shorter wavelength, requiring the light source to be hard X‐rays or high energy (tens of keV) electrons. Time‐resolved X‐ray diffraction using incoherent plasma sources can disclose dynamic information on atomic and subatomic scales. The shortest pulses produced by synchrotrons can reach a duration of tens of picoseconds. X‐ray free electron lasers, especially Linac Coherent Light Source (such as LCLS I in the USA, SACLA in Japan, FERMI in Italy, etc.), offer a significant improvement in time resolution, opening access to structural dynamics detection under femtosecond scale.^[^ ^162^ ^]^

*Refinement of the DFT Approaches*: The approximated exchange‐correlation function used in DFT calculations causes deviations in the total electronic energy of the adsorbed substances.^[^ ^163^ ^]^ The parameters obtained from periodic boundary conditions inherently have an error between 0.1 and 0.2 eV. Ongoing modifications to the approximate exchange‐correlation functionals include additions of spin kinetic energy densities (meta‐GGA) and the hybrid of the non‐local Hartree‐Fock exchange part to approach the virtual orbitals for the exchange‐correlation energy, thus enabling more accurate energy calculation.^[^ ^163^ ^]^ Additionally, several important effects are often not considered in DFT, such as the influence of co‐adsorbed species on the binding energies, the co‐existence of differing facets, and the dynamic restructuring (or fluxional state) of the catalysts during the reaction. Specifically for electrochemical systems, DFT calculations are usually carried out in a vacuum and do not account for factors such as the solvation effects, water network, electric field effects, mass transfer, etc.^[^ ^15^ ^]^

*Dynamic Evolution of Heterostructured Electrocatalysts*: Current research on heterostructures used as electrocatalysts mainly considers redistribution of valence electrons without potential bias, and its impact on the adsorption energies of intermediates. However, there is little attention given to the rectification effect and its role in accelerating the electrochemical process. Pioneering studies, such as the work of Li's group on the *p‐n* junction of FePc‐GaS, have specified the role of the rectification effect of heterojunction in accelerating the electrocatalytic process.^[^ ^76^ ^]^ However, the influence of concentration of the number of charge carriers, work function, and conductivity on the rectification effect under potential bias, and their impact on the binding energy of adsorbates are still rarely addressed. On the other hand, while the built‐in electric field can be deduced through the determination of the Zeta potential or by performing DFT calculations, physical characterizations are still lacking to capture its dynamic evolution under bias. This makes it difficult to study the role of the amplified built‐in electric field on the Helmholtz layer, the surface hydrogen bond network, and the mass transfer. Ma and coworkers^[^ ^164^ ^]^ revealed the relation between the gap of work functions (∆Φ) for PtM /CoP (M  =  Rh, Pd, Ag, Ir, and Au) heterostructures and the hydrogen spillover kinetics, and found the most effective hydrogen spillover for the combination of PtIr/CoP with close work functions. They specified that smaller ∆Φ not only induced interfacial charge relocation, but also impaired interfacial proton adsorption and ensured efficient hydrogen spillover for HER. However, the role of the built‐in electric field under bias on the overflow of adsorbed hydrogen at the interface remains to be clarified.

In recent research on heterostructures, the focus has been mostly on the determination of the electrostatic interaction of electrons, but interactions with adsorbates and their influence on the lattice of the catalysts have been often overlooked. Specifically, for electrocatalysts under operating conditions, the surface usually undergoes a reconstruction through interactions with electric fields, electrolytes, intermediates, and reactants. The complex composition of the reconstructed electrocatalysts can induce additional challenges in exploring electrocatalytic mechanisms and identifying the actual catalytic sites. Thus, various *operando* or ex situ characterizations, such as femtosecond time‐resolved transient absorption spectroscopy, in situ electron microscopy, scalable X‐ray absorption fine‐structure techniques, and grazing incidence X‐ray diffraction are required to understand the evolution of the catalysts in the reconstruction process and to guide the design of optimized catalysts.

*Machine Learning*: The complexity of heterostructured electrocatalysts and the large variety of molecules involved in catalytic reactions result in a large number of possible combinations, making it challenging even for high‐throughput experimentation and computational analysis to arrive at the most optimal design. With advancement in physical characterizations and supercomputers, large volumes of experimental and simulated data become available. Those databases allow the use of artificial intelligence and machine learning to identify hidden patterns and correlations in bridging the experiment‐theory gaps. For instance, by combining microscopic, spectroscopic, and machine learning molecular dynamics, microsecond and atomic scales of simulations of the restructuring process of Pd on Ag can be reached.^[^ ^165^ ^]^ Machine learning algorithms can also alleviate the issue of accuracy‐efficiency trade‐off in describing the exchange–correlation effects of many‐electron systems in ab initio simulations, thereby enhancing the accuracy of transition state energy and speeding up the computational modeling of active sites. Another significant application of machine learning is in enhancing the analysis of experimental data, including microscopic, spectroscopic, and kinetic data related to the electrocatalysts.

## Conflict of Interest

The authors declare no conflict of interest.
